# Measuring the Coupling Direction between Neural Oscillations with Weighted Symbolic Transfer Entropy

**DOI:** 10.3390/e22121442

**Published:** 2020-12-21

**Authors:** Zhaohui Li, Shuaifei Li, Tao Yu, Xiaoli Li

**Affiliations:** 1 School of Information Science and Engineering (School of Software), Yanshan University, Qinhuangdao 066004, China; lizhaohui@ysu.edu.cn (Z.L.); lishuaifei@stumail.ysu.edu.cn (S.L.); 2 Hebei Key Laboratory of Information Transmission and Signal Processing, Yanshan University, Qinhuangdao 066004, China; 3 Beijing Institute of Functional Neurosurgery, Capital Medical University, Beijing 100053, China; yutaoly@sina.com; 4 State Key Laboratory of Cognitive Neuroscience and Learning, Beijing Normal University, Beijing 100875, China

**Keywords:** neural oscillation, coupling direction, symbolic transfer entropy, weighted probability distribution, epileptic seizure

## Abstract

Neural oscillations reflect rhythmic fluctuations in the synchronization of neuronal populations and play a significant role in neural processing. To further understand the dynamic interactions between different regions in the brain, it is necessary to estimate the coupling direction between neural oscillations. Here, we developed a novel method, termed weighted symbolic transfer entropy (WSTE), that combines symbolic transfer entropy (STE) and weighted probability distribution to measure the directionality between two neuronal populations. The traditional STE ignores the degree of difference between the amplitude values of a time series. In our proposed WSTE method, this information is picked up by utilizing a weighted probability distribution. The simulation analysis shows that the WSTE method can effectively estimate the coupling direction between two neural oscillations. In comparison with STE, the new method is more sensitive to the coupling strength and is more robust against noise. When applied to epileptic electrocorticography data, a significant coupling direction from the anterior nucleus of thalamus (ANT) to the seizure onset zone (SOZ) was detected during seizures. Considering the superiorities of the WSTE method, it is greatly advantageous to measure the coupling direction between neural oscillations and consequently characterize the information flow between different brain regions.

## 1. Introduction

Neural oscillations are rhythmic patterns of electrical activity produced by the interaction of neurons in the nervous system [1], which are the fundamental mechanism to achieve coordinated activities in the brain [2,3,4]. The oscillations are ubiquitously observed in mammalian brains and involved in a variety of brain operations, including visual attention [5,6], memory formation [7,8], and stimulation processing [9,10]. Although it is relatively easy to observe the modulation of neural oscillations, what role they play in neural information processing is still unclear [11,12,13]. A noteworthy feature of neural oscillations is the specific coupling between different frequency rhythms [14,15]. The coupling between neural oscillations is of crucial importance in the communication across areas of the brain and the integration of information [16,17]. It may take a variety of forms, appear in different areas, and support distinct functions [18,19]. The integrated functions of the brain, such as vision and hearing, require the cooperation of several spatially separated brain regions [20,21]. Therefore, a reliable estimate of this cooperation is essential to reveal the functional connectivity between brain regions. Many methods have been introduced to analyze electrophysiological recordings and quantify the coupling strength between neural oscillations in different brain regions [22], such as likelihood synchronization [23], coupling analysis [24], and nonlinear interdependence measures [25]. However, most of them focus on the strength of pairwise interactions (i.e., the degree of similarity or dissimilarity between two neural oscillations).

In fact, the coupling direction is also of great significance to characterizing the interaction between neuronal oscillations [26]. One of the frequently used methods is the Granger causality, which evaluates the directionality by measuring the effect of historical information of one time series on the prediction error of future moments in another time series [27,28,29,30]. According to previous studies, the Granger causality method is statistically significant and can be successfully applied to linear models, but it only gives qualitative analysis results and cannot be applied to nonlinear models directly [31]. Other prevailing methods used to estimate the coupling direction are mostly based on information theory, such as conditional mutual information [32] and transfer entropy [33,34]. These methods measure the information provided by the sourcing process on the state transitions in the target process, characterizing the non-linear causality between time series [35]. However, they generally require a large amount of data and are prone to be affected by the noise. Therefore, a technique of symbolization based on phase space reconstruction and permutations has been applied to improve the performance of these information-based methods. Typical examples are permutation conditional mutual information [36,37] and symbolic transfer entropy [38,39]. Considering that the construction of a symbolized sequence ignores the amplitude information in the original data and reduces the impact of larger fluctuations in the time series on the results [40,41,42,43], we used weighted probability to calculate the joint distribution of the permutation motifs and consequently obtain the symbolic transfer entropy based on the weighted probability distribution. The proposed method to measure the coupling direction is termed weighted symbolic transfer entropy (WSTE) in this study. We compared WSTE and STE by using a neural mass model, and the results of the new method showed that WSTE was significantly better than STE at detecting the directionality index, even in noisy data. Moreover, to test the validity, we used the proposed method to analyze the epileptic electrocorticography (ECoG) recorded from a patient with epilepsy. The results indicated a significant coupling direction from the thalamus to the cortex during seizures.

The remainder of this paper is organized as follows. Section 2 introduces the definitions of STE and WSTE. Section 3 compares the performance of STE and WSTE based on the neural oscillations model and uses WSTE to estimate the coupling direction between the thalamus and the seizure onset zone. Finally, we conclude our paper in Section 4.

## 2. Materials and Methods

### 2.1. Symbolic Transfer Entropy (STE)

Symbolic transfer entropy adopts a technique of symbolization, which has also been introduced with the definition of permutation entropy [38,44]. Suppose a time series $X\;=\;{\left\{x_{t}\right\}}_{t=1}^{T}\;$ is recorded from a certain area of the brain for an arbitrary *i*. *X* can be reconstructed into the time delay embedding representation $X_{i}^{m,\tau }$, as shown in Equation (1):

(1) $$X_{i}^{m,\tau }\;=\;\;\left\{x_{i},x_{i+\tau },\ldots \ldots ,x_{i+(m-1)\tau }\right\},\;\;i=1\;,\;2\;,\;\ldots \;,\;T-(\;m-1\;)\;\tau ,$$

where *m* and *τ* denote the embedding dimension and time delay, respectively. To get the sequence of symbols, each vector is arranged in ascending order of the elements:

(2) $$x_{i+(l_{1}-1)\tau }\leq x_{i+(l_{2}-1)\tau }\leq \cdots \leq x_{i+(l_{m}-1)\tau },$$

The original index of elements forms a sequence $\left\{l_{1},l_{2},\cdots ,l_{m}\right\}$, representing one of *m*! possible permutations, which can be mapped onto a corresponding symbol $\pi_{k}$, k=1,2,⋯,m!. For example, if *m* = 3, the following six ordinal patterns and their corresponding symbols are $\{1,2,3\}\to \pi_{1}\;,\;$$\{1,3,2\}\to \pi_{2},\;$$\{2,1,3\}\to \pi_{3},\;$$\{2,3,1\}\to \pi_{4},\;$$\{3,1,2\}\to \pi_{5},\;$$\{3,2,1\}\to \pi_{6},\;$ and if $X_{i}^{3,1}=\{1.26,-0.45,0.81\}$, the symbol of $X_{i}^{3,1}$ will be $\pi_{4}$. Let S(⋅) denote the map from the ordinal pattern space to the symbol space. Each $\;X_{i}^{m,\tau }\;$ is uniquely mapped onto one of the *m*! possible symbols such that $S(X_{i}^{m,\tau })=\pi_{k}$. Therefore, the symbol sequences are given by

(3) $$\hat{x}=\left\{S(X_{1}^{m,\tau }),S(X_{2}^{m,\tau }),\cdots ,S(X_{T-(m-1)\tau }^{m,\tau })\right\},$$

(4) $$\hat{y}=\left\{S(Y_{1}^{m,\tau }),S(Y_{2}^{m,\tau }),\cdots ,S(Y_{T-(m-1)\tau }^{m,\tau })\right\},$$

Another symbol sequence, $\;{\hat{x}}_{\delta }\;$ is delayed by *δ* steps form $\hat{x}$:

(5) $${\hat{x}}_{\delta }=\left\{S(X_{1+\delta }^{m,\tau }),S(X_{2+\delta }^{m,\tau }),\cdots \right\},$$

The symbolic transfer entropy (STE) from *Y* to *X* can be defined as [38]

(6) $$T_{Y\to X}^{\delta }=\sum_{\hat{x},\hat{y}}p\left({\hat{x}}_{\delta },\hat{x},\hat{y}\right)\log\frac{p\left({\hat{x}}_{\delta }|\hat{x},\hat{y}\right)}{p\left({\hat{x}}_{\delta }|\hat{x}\right)},$$

The information entropy that is transferred from $\hat{y}$ to $\hat{x}$ at some later points in time can be defined as [45]

(7) $$T_{Y\to X}\;=\;T_{Y\to X}^{\delta^{\prime }}\;,\;\;\delta^{\prime }=\;\arg\;\max(T_{Y\to X}^{\delta }),$$

In this equation, $T_{X\to Y}$ is defined in a complete analogy. Finally, the directionality index between *X* and *Y* is given by [46]

(8) $$D^{S}=T_{X\to Y}-T_{Y\to X}$$

The directionality index $D^{S}$ quantifies the direction of information flow between two neural oscillations. It is positive if the direction of information flow is from *X* to *Y*; if it is the contrary, then it is negative.

Obviously, the definition of STE involves probability calculations, which is the focus of this section. It is generally considered that the probability for the occurrence of a symbol $\;p\left(\pi_{k}\right)\;$ is equivalent to the proportion of the symbol in the finite sequence. Thus, it can be calculated as follows:

(9) $$p\left(\pi_{k}\right)=\frac{\sum_{i=1}^{T-(m-1)\tau }I\left\{S(X_{i}^{m,\tau }),\pi_{k}\right\}}{T-(m-1)\tau },$$

where k=1,2,…,m!, $I\left\{S(X_{i}^{m,\tau }),\pi_{k}\right\}=1$ if $X_{i}^{m,\tau }$ can map onto symbol $\pi_{k}$; otherwise $I\left\{S(X_{i}^{m,\tau }),\pi_{k}\right\}=0$.

**Figure 1:**
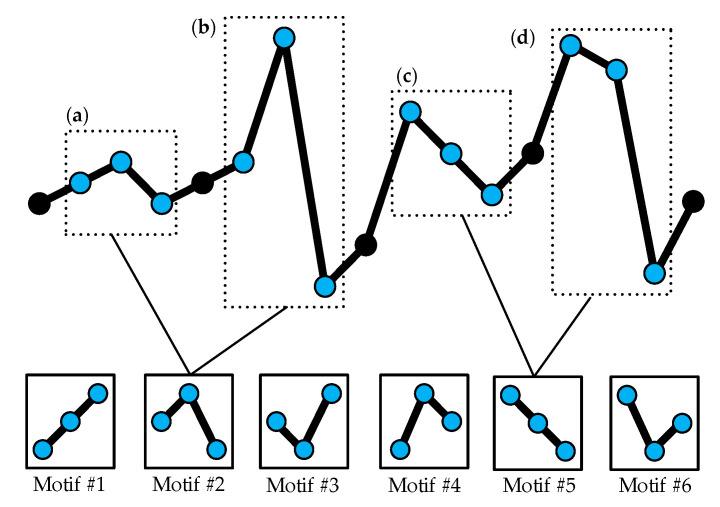
Two examples of different vectors (*m =* 3, *τ =* 1) in a time series mapped onto the same motif. Six motifs are available in total. (**a**–**d**) Examples for Motif #2 and Motif #5.

The main disadvantage of the above definition for the symbols is that it only focuses on the sequential structure and ignores the degree of difference between the amplitude values of the time series. Therefore, the influence of large fluctuations in the time series on the final results of the transfer entropy is also not considered. On the other hand, the small fluctuations which may be induced by noise should not be supposed to exert the same influence on the final STE value as the large fluctuations. Figure 1 demonstrates that different m-dimensional vectors are treated as the same ordinal pattern. Obviously, although (a) and (b) have a great difference in amplitude, they are regarded as the same order pattern. Another example is (c) and (d). Thus, it is necessary to emphasize the degree of difference between the amplitudes.

### 2.2. Weighted Symbolic Transfer Entropy

In this section, the weighted probability is introduced into the STE, which has been applied in weighted permutation entropy and can improve the robustness of the algorithm against noise [40]. For a symbol $\pi_{k}$, the weighted probability $p_{w}(\pi_{k})$ can be calculated:

(10) $$p_{w}\left(\pi_{k}\right)=\frac{\sum_{i=1}^{T\;-\;(m-1)\tau }I\left\{S(X_{i}^{m,\tau }),\pi_{k}\right\}\cdot w_{i}}{W},$$

where $\;w_{i}\;$ is the standard deviation of $X_{i}^{m,\tau }$, indicating the weight value of the *i*th symbol in $\hat{x}$:

(11) $$w_{i}=\sqrt{\frac{1}{m}\sum_{j=1}^{m}{\left(x_{i+\left(j-1\right)\tau }-{\bar{X}}_{i}^{m,\tau }\right)}^{2}},$$

where *W* will be the new denominator instead of T−(m−1)τ:

(12) $$W=\sum_{i=1}^{T-(m-1)\tau }w_{i}\;.$$

The degree of difference between motifs with different amplitudes can be highlighted by the weight $w_{i}$. For the given signals *X* and *Y* and their reconstructed matrices $X^{m,\tau }$ and $Y^{m,\tau }$, we get the symbol combinations $\left(\pi_{k}^{x},\pi_{k}^{y}\right)$ such that *X* and *Y* are mapped at the same time, and there are ${\left(m!\right)}^{2}$ symbol combinations for *X* and *Y*. The weighted joint probability can be calculated as

(13) $$p_{w}\left(\pi_{k}^{x},\pi_{k}^{y}\right)=\frac{\sum_{i=1}^{T-(m-1)\tau }I\left\{S(X_{i}^{m,\tau },Y_{i}^{m,\tau }),(\pi_{k}^{x},\pi_{k}^{y})\right\}\cdot w_{i}^{x}\cdot w_{i}^{y}}{W^{x}\cdot W^{y}},$$

where the definitions of $w_{i}^{x},w_{i}^{y}$ and $W^{x},W^{y}$ are the same as above.

Therefore, WSTE can be defined as

(14) $$WT_{Y\to X}^{\delta }\;=\;\sum_{\hat{x},\hat{y}}p_{w}\left({\hat{x}}_{\delta },\hat{x},\hat{y}\right)\log\frac{p_{w}\left({\hat{x}}_{\delta }|\hat{x},\hat{y}\right)}{p_{w}\left({\hat{x}}_{\delta }|\hat{x}\right)},$$

Similar to Equations (7) and (8), the directionality index of WSTE between *X* and *Y* is given by

(15) $$D^{W}\;=WT_{X\to Y}^{}\;-\;\;WT_{Y\to X}^{}.$$

#### 2.2.1. Parameters of WSTE

There are three parameters in the calculation of WSTE, including the embedding dimension m, time delay *τ*, and *δ*. The embedding dimension m is the number of points in each permutation pattern. Generally, the recommended values are *m* = 3…7 [44]. If *m* = 1 or 2, very few permutation patterns are included, which makes the method meaningless. It is acceptable to choose a larger m for the analysis of long stationary time series. However, for two neural signals, each with a sampling length *L*, *m* has to satisfy the condition $\;L>{\left(m!\right)}^{3}$ so as to ensure the occurrence of every possible joint permutation pattern. Taking *m* = 4 as an example, the sample length of the data *L* should be greater than $\;{\left(4!\right)}^{3}=\;13824$. In short, to obtain the temporal characteristics of neural signals, a large value of *m* is not appropriate. Thus, *m* = 3 was selected for the calculation of WSTE in the following analyses.

**Figure 2:**
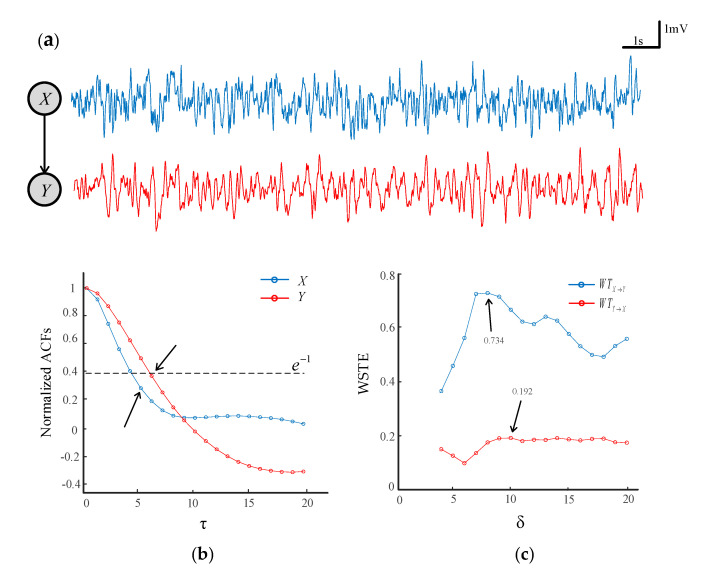
The determination of the lag base on the autocorrelation function. (**a**) Two simulated time series with *X* driving *Y*. (**b**) The normalized autocorrelation functions (ACFs) of time series *X* and *Y*. (**c**) The weighted symbolic transfer entropy (WSTE) between *X* and *Y* with different *δ*.

The lag *τ* is referred to as the number of sample points spanned by each permutation pattern, which is associated with the sampling frequency of a time series. The higher the sampling frequency, the longer the lag *τ*. In practice, we could employ an autocorrelation function (ACF) of a signal to determine the lag *τ*. Figure 2a plots two time series generated by a coupled neural mass model (see Section 2.3). Figure 2b shows the ACFs of time series *X* and *Y* that are normalized to unify at zero lag. An optimal lag could be determined at the point where the ACF had decayed to $\;e^{-1}\;$ of its peak value [47]. It can be seen that the ACF was less than $e^{-1}$ when the lag *τ* = 5 and *τ* = 6 for time series *X* and *Y*, respectively. As such, in this case, the lag *τ* = 6 could be selected for calculating the WSTE.

The *δ* is related to the delay between time series *X* and *Y*, and *δ* cannot be less than the embedding dimension m in the WSTE calculation [32]. Here, we made the *δ* range from 3 to 20. Given *m* = 3 and *τ* = 6, the transfer entropy at different *δ* is plotted in Figure 2c. It is shown that there was a clear maximum of $WT_{X\to Y}$ at *δ* = 8, which indicates the delay of information transferred from *X* to *Y*. On the contrary, the $WT_{Y\to X}$ was relatively small, and there was no obvious difference for the values of *δ*, meaning that no coupling was available in the direction from *Y* to *X*.

#### 2.2.2. Significance of WSTE

In this study, we used a surrogate method [48] to test the significance of the directionality index. The control group was a set of surrogate data obtained by randomly shuffling the original time series [33]. The surrogate data retained the same distribution, but the random shuffling destroyed the ordinal patterns of the original time series. Thus, the surrogate data could be regarded as independent, and the motifs constructed from the original series changed correspondingly. If there was a significant difference in the direction index between the original data and the surrogate data, the null hypothesis (two series are independent) should have been rejected.

For each original series, 100 surrogate data were generated to test the statistically significant difference of the directionality index between the original and surrogate series. The range of the mean ± 2 * SD (standard deviations) of the surrogate directionality index without any coupling direction could be used to illustrate its fluctuation. If the directionality of the original data was located within this range, it would be considered insignificant and set to zero. The processed directionality index was called the filtered directionality index.

### 2.3. Coupled Neural Mass Model

To evaluate the performance of the WSTE algorithm, it was necessary to use an appropriate model of reference for practical applications in complex systems. A neural mass model (NMM) [49,50] was employed to generate the coupled neural oscillations to test the performance of our proposed WSTE method. Based on the average field modeling idea, the model reflects the average behavior of the whole neuronal population in the neural networks with lumped state variables. The model constructs the neural oscillations from the perspective of the tissue structure in the nervous system, which is simple and physiological. The coupled NMM can reflect the interrelationship between neuron groups and simulate large-scale interactive neural networks at a macro level [51]. The NMM is represented by Equation (16).

The parameters in the model were physiologically realistic, and their details are listed in Table 1. In the model, the internal behavior of the neuronal population was mainly affected by the excitatory neuron parameter *A* and the inhibitory neuron parameter *B*. In Section 3.1, we set $A_{1}=A_{2}=3.25$ and $B_{1}=B_{2}=35$ for two oscillators. The static nonlinear function *S* is represented by the sigmoid function $S(v)=2e_{0}/(1+e^{r(v_{0}-v)})$. The extrinsic input $P^{\left(t\right)}$ represents Gaussian white noise, with an assigned mean value and variance. This describes the overall density of action potentials coming from other regions and is similar to subcortical–cortical excitatory drive. Another important parameter is the connection strength *K*, which defines the degree and direction of coupling between neuronal populations [52,53]. In this study, initial conditions were set to zero in all simulations, and an integration step size of 5 ms (sampling frequency of 200 Hz) was used. Two coupled neuronal populations were generated, and the effect of the parameters on the directionality index were examined.

(16) $$\left\{ \begin{array}{l} {\dot{y}}_{0}^{n}(t)=y_{3}^{n}(t),\\ {\dot{y}}_{3}^{n}(t)=AaS\left[y_{1}^{n}(t)-y_{2}^{n}(t)\right]-2ay_{3}^{n}(t)-a^{2}y_{0}^{n}(t),\\ {\dot{y}}_{1}^{n}(t)=y_{4}^{n}(t),\\ {\dot{y}}_{4}^{n}(t)=Aa\left\{p^{n}(t)+C_{2}S\left[C_{1}y_{0}^{n}(t)\right]+\sum_{i=1,\ldots ,N,i\neq n}K^{i}y_{6}^{i}(t)\right\}-2ay_{4}^{n}(t)-a^{2}y_{1}^{n}(t),\\ {\dot{y}}_{2}^{n}(t)=y_{5}^{n}(t),\\ {\dot{y}}_{5}^{n}(t)=Bb\left\{C_{4}S\left[C_{3}y_{0}^{n}(t)\right]\right\}-2by_{5}^{n}(t)-b^{2}y_{2}^{n}(t),\\ {\dot{y}}_{6}^{n}(t)=y_{7}^{n}(t),\\ {\dot{y}}_{7}^{n}(t)=Aa_{d}S\left[\left(y_{1}^{n}(t)-y_{2}^{n}(t)\right)\right]-2a_{d}y_{7}^{n}(t)-a_{d}^{2}y_{6}^{n}(t), \end{array}\right.\;\;n=1,2$$

### 2.4. Epileptic ECoG Data

Investigating the spread of epilepsy discharges can reveal whether there is an interaction between the seizure onset zone and the remote area [54]. For instance, if a seizure starts in one area of the brain, then it may cause another region to follow it by a coupling [55,56,57]. Detecting the coupling direction between different brain regions can help to make a deep understanding of the propagating mechanism for seizures and the structure of epileptic networks [58].

**Figure 3:**
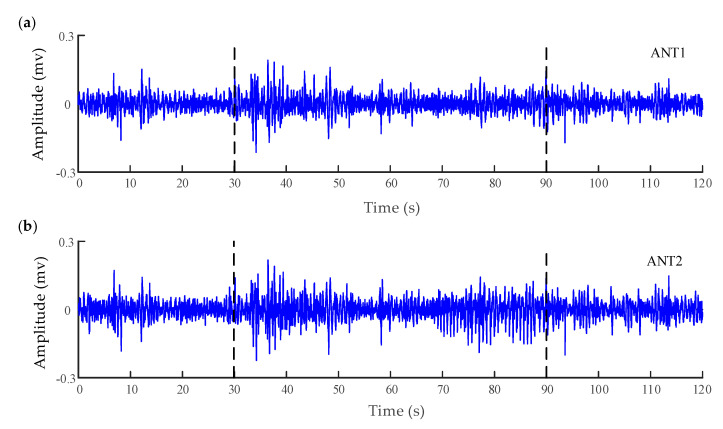

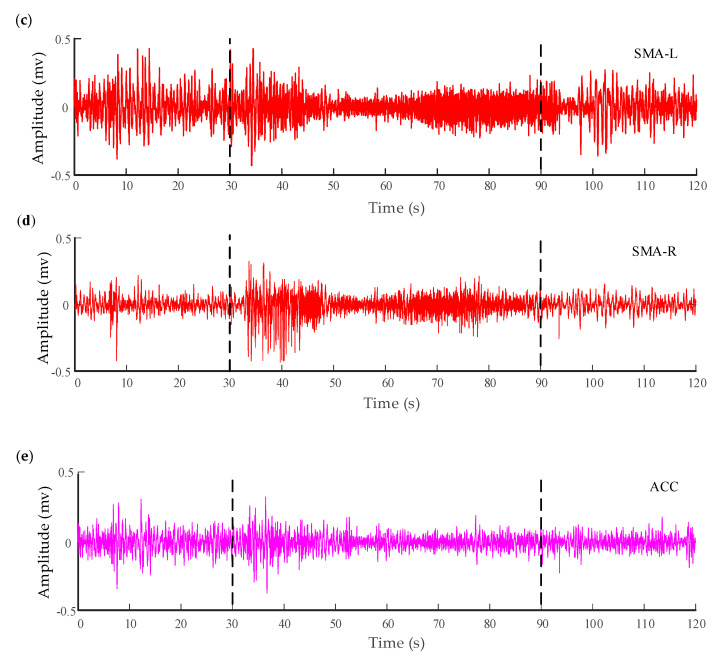
Electrocorticography (ECoG) of six channels recorded from a patient with a refractory focal seizure. (**a**,**b**) are the recordings in the anterior nucleus of thalamus (ANT). (**c**) The recordings in the left supplementary motor area (SMA). (**d**) The recordings in the right SMA (epilepsy focus). The vertical lines in these panels indicate the beginning and termination of the seizure. (**e**) The recordings in the anterior cingulate cortex (ACC) (the normal cortex without seizures).

To characterize the interaction between the different brain regions of a patient (male, 18 years old) with refractory focal seizures, the WSTE was used to estimate the coupling direction of the recorded electrocorticography (ECoG). The depth electrodes were semi-rigid platinum/iridium with 16 contacts. One of the clinical depth electrodes exploring the frontal cortex, or the peri-insular cortex, was extended into the anterior nucleus of thalamus (ANT) after a subtle angle adjustment. The patient had signed informed consent that this clinical data might be used for research purposes, and the study protocol had previously been approved by the local ethics committee. The raw signals were recorded by a Micromed electroencephalogram (EEG) data acquisition system with a sampling frequency of 1024 Hz, referencing a common contact placed subcutaneously. All surgical and electrophysiological records were performed at the participating hospital (Xuanwu Hospital Capital Medical University). More details can be found in [59]. Before calculating the directionality index, the recorded signals were filtered at 0.5–100 Hz with a Butterworth band-pass filter, and an adaptive notch filter was used to remove the 50 Hz power signal. Prior to analysis, the processed data had been down-sampled to 512 Hz. A segment of 120 s which involved a seizure was extracted from the whole recording. The down-sampled signals of six channels are demonstrated in Figure 3, which were located in the anterior nucleus of thalamus (ANT), supplementary motor area (SMA), and anterior cingulate cortex (ACC). The seizure started at approximately 30 s in the recording and lasted about 60 s.

## 3. Results

### 3.1. Application to the Neural Mass Model

In simulation analysis, the bionic nonlinear model was adopted because of its consistency with the main idea of this study. First, the basic principle and parameters of the neural mass model (NMM) are presented. Secondly, we measured the sensitivity of WSTE’s variation with the coupling strength of the model. Finally, by comparing this with the traditional STE method in the aspects of data length and noise immunity, the superiority of WSTE is illustrated.

#### 3.1.1. Variation of the Directionality Index with Coupling Strength

**Figure 4:**
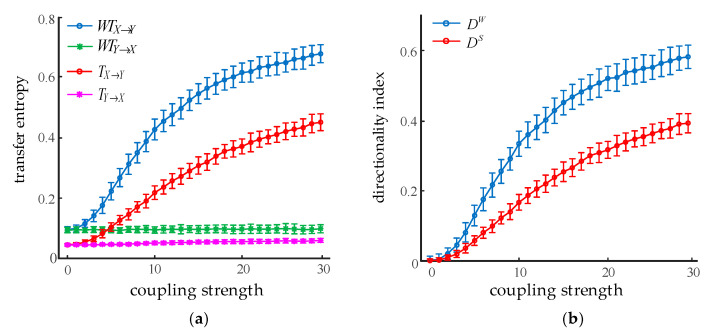
Effect of coupling strength on STE and WSTE. (**a**) Transfer entropy in two directions. (**b**) Directionality index from *X* to *Y*.

Given the coupling strength $K_{1,2}=0$ and its increasing from 0 to 50 in steps of 1, two oscillations with a length of 20 s were generated. We used *m =* 3 and *τ =* 6 to calculate the directionality index between the oscillations with WSTE and STE, respectively. As shown in Figure 4, the results are given in the form of mean ± SD of 100 realizations for each method. Figure 4a plots the dependence of transfer entropy on the coupling strength $K_{2,1}$. As $K_{2,1}$ increased from 0 to 30, both $T_{Y\to X}$ and $WT_{Y\to X}$ did not change significantly. However, in the opposite direction, the values of $T_{X\to Y}$ and $WT_{X\to Y}$ were continuously growing, which was consistent with the direction of information flow from *X* to *Y*. Figure 4b demonstrates the relationship between the directionality index and the coupling coefficient. As the coupling coefficient $K_{2,1}$ changed from 0 to 30, both the directional indexes kept increasing. In addition, it is worth noting that the values of $WT_{X\to Y}$ were always larger than $WT_{Y\to X}$, and the values of $D^{W}$ were significantly higher than $D^{S}$ (ANOVA, *p* < 0.001) while the coupling strength was more than 4. This suggests that the WSTE method was more effective and appropriate for detecting the coupling strength between two oscillations.

#### 3.1.2. Robustness of the Directionality Index Against Noise

**Figure 5:**
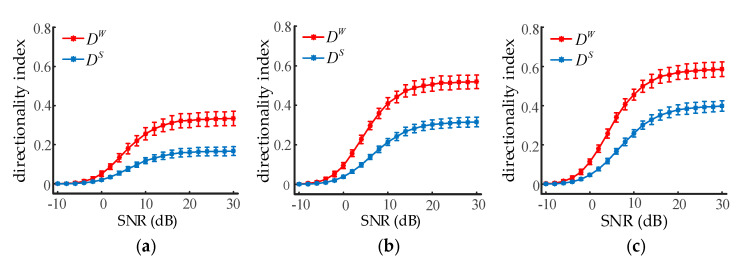
The influence of noise on the directionality indexes of STE and WSTE. (**a**) The coupling strength $K_{2,1}$ is set to 10. (**b**) The coupling strength $K_{2,1}$ is set to 20. (**c**) The coupling strength $K_{2,1}$ is set to 30.

Adding measurement noise into the signals can assess the robustness of the directionality index without disturbing the inherent dynamics of the system. Given $K_{1,2}=0$, we set $K_{2,1}$ to 10, 20, and 30, respectively, and two neuronal population outputs of 20 s were generated. The signal-to-noise ratio (SNR) ranged from −10 dB to 30 dB with a step of 1 dB. The effect of white Gaussian noise on the directionality index at three different coupling strengths is illustrated in Figure 5. Error bars indicated the average and standard deviations from 100 trials of calculations. As can be seen from the results, both $D^{S}$ and $D^{W}$ were deteriorated severely by the noise when the SNR was less than about 10 dB. However, it was also found that $D^{W}$ was significantly higher than $D^{S}$ when the SNR was greater than −2 dB (ANOVA, *p* < 0.001). Thus, it is reasonable to conclude WSTE was superior to STE at identifying the coupling direction between two neural oscillations.

#### 3.1.3. Variation of the Directionality Index with Sample Length

**Figure 6:**
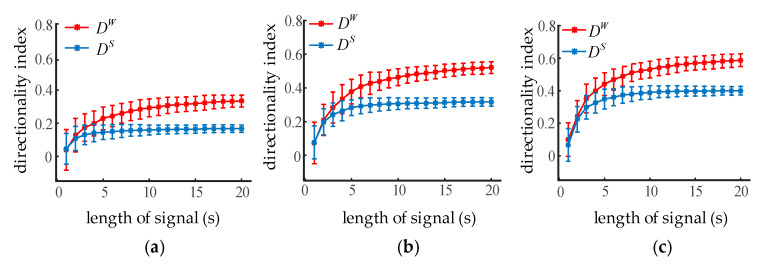
The influence of sample length on the directionality index of STE and WSTE. (**a**) The coupling strength $K_{2,1}$ is 10. (**b**) The coupling strength $K_{2,1}$ is 20. (**c**) The coupling strength $K_{2,1}$ is 30.

Transfer entropy depends on the statistical calculation of the conditional mutual information; therefore, the influence of the sample length on the directionality index should be investigated. Given $K_{1,2}\;=0$, $K_{2,1}$ increased from 10 to 30 in steps of 10, the NMM output oscillations with lengths from 1 s to 20 s in steps of 1 s. The directionality index of the simulated oscillations for each coupling strength was calculated. Error bars indicated the mean and standard deviations from 100 trials. It can be seen from Figure 6 that the average directionality index increased with the sample length, while the standard deviation decreased at the same time. The blue curve is stable when the sampling length exceeds 7 s, while the red one is still growing slightly. STE and WSTE could both reliably distinguish the coupling direction between two neural oscillations when the sample length was greater than 2 s (400 sampling points). Moreover, the $D^{W}$ was larger than the $D^{S}$ while the sample length was over 8 s, indicating that WSTE performed better than STE with enough data points.

### 3.2. Application to Epileptic Seizures

**Figure 7:**
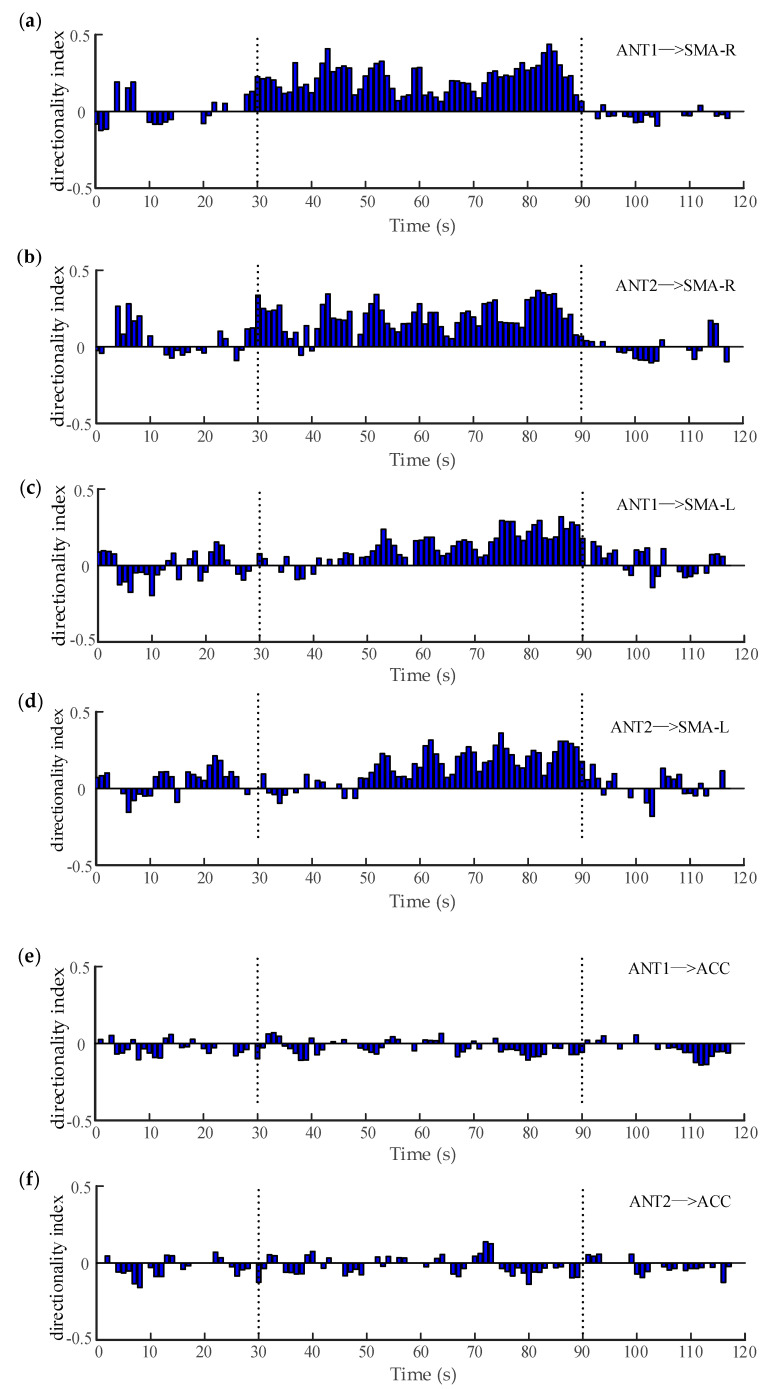
The variation of the directionality index between different regions. (**a**,**b**) are the directionality index between the ANT and the right SMA. (**c**,**d**) are the directionality index between the ANT and the left SMA. (**e**,**f**) are the directionality index between the ANT and the ACC.

A window of 3 s with an overlap of 2 s was utilized to calculate the significance of the directionality index. Figure 7a,b illustrate the variation of the filtered coupling directionality index between ANT and right SMA (epilepsy focus) over time. It can be seen that the directionality index between the ANT and right SMA was mostly positive and significant during the seizure (30–90 s), indicating the information transferred from the ANT to the right SMA. In other words, the ANT exerted some driving effects on the epilepsy focus, which may have played an important role in promoting or maintaining the abnormal activity of the epileptic network. Actually, deep brain stimulation (DBS) has emerged as an effective form of therapy for drug-resistant epilepsy [60]. Among a number of brain targets, the ANT is generally considered as a potential target because of its central connectivity and possible role in the propagation of seizure activity [59]. Thus, it is proven that our results are consistent with previous studies. Similarly, Figure 7c,d demonstrates the variation of the directionality index between the ANT and the left SMA over time. It was shown that there were no clear unidirectional couplings between the two regions in the earlier period of the seizure (30–50 s), whereas the directionality index between the ANT and the left SMA were mostly positive during the later period of the seizure (50–90 s). This means that the driving relationship from the ANT to the left SMA was also significant, but delayed by approximately 20 s, which will be further analyzed in our future works. On the other hand, when the ANT and the ACC entered the computation of WSTE, there was no similar phenomenon observed between these two regions. As shown in Figure 7e,f, even during the onset interval, the relatively weak coupling appeared in both directions. This suggests that there is no obvious causal relationship available from the ANT to the ACC and, consequently, the seizure does not occur in the ACC.

### 3.3. Comparison between WSTE and STE with Noisy Experimental Data

**Figure 8:**
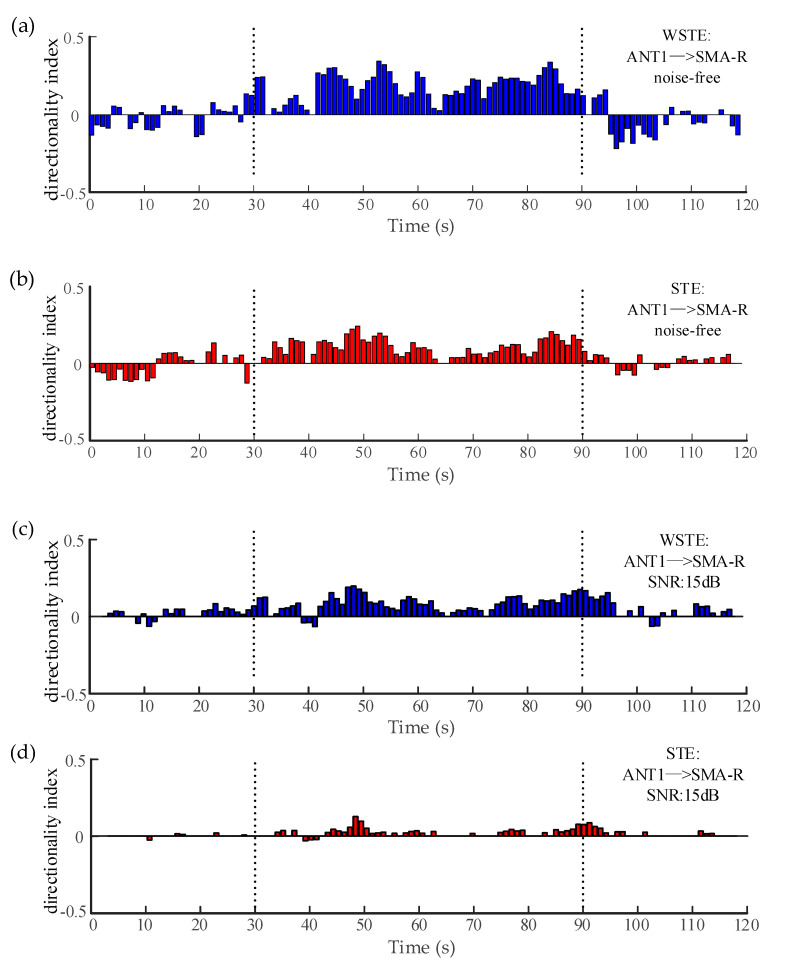

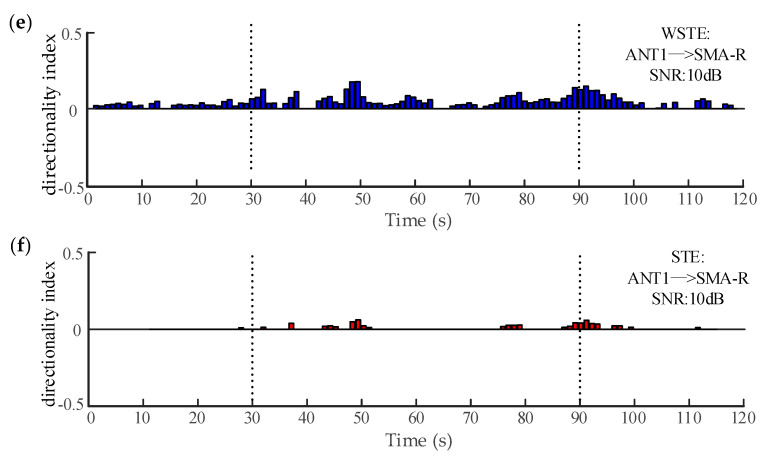
The variation of the directionality index between the ANT and the right SMA. (**a**,**b**) show WSTE and STE without noise. (**c**,**d**) show WSTE and STE for a 15 dB signal-to-noise ratio (SNR). (**e**,**f**) show WSTE and STE for a 10 dB SNR.

The electrocorticography in Section 2.4 and Section 3.2 can be considered noise-free. Figure 8a,b plots the directionality indices of the two algorithms when analyzing the noise-free data, and both could estimate significant directionality indices during the seizure (30–90 s). However, WSTE outperformed STE in noisy time series. To evaluate the robustness against noise, we added white Gaussian noise to the time series of ANT1 and SMA-R before calculating the directionality index. Clearly, as shown in Figure 8c–f, WSTE could still give significant directionality indices during the seizure, while STE could hardly detect the coupling direction, especially when the SNR was lower than 10 dB.

## 4. Conclusions

The brain is a complex non-linear physiological system. The coupling direction between neural oscillations is of great importance to studying brain functions. The transfer entropy algorithm is an effective tool to estimate the coupling direction based on information theory, which establishes a causal relationship between neural oscillations. In this study, we proposed a novel WSTE method to analyze the coupling direction between two neural oscillations. By estimating the weighted probability distribution, WSTE emphasizes the degree of difference between the amplitudes of a time series while defining symbols. In contrast, this information is ignored in the calculation of STE. The results of simulated data show that WSTE is superior to the traditional STE method, particularly in the sensitivity of coupling strength, the requirement of data length, and the performance against noise. The proposed WSTE method is also used to analyze the experimental recordings of an epilepsy patient. It was found that there was a significant coupling direction from the ANT to the seizure onset zone (SOZ) during the epileptic seizure, which is consistent with some conclusions about deep brain stimulation. In the application of human data, both WSTE and STE could describe the spatial connection and causal relationship between different brain areas. Although they could give consistent results in the noiseless case, WSTE was more robust than STE when the noise was available. To conclude, our main work is to optimize the STE method and improve its performance, and we suggest that the novel measure is a powerful and effective tool for estimating the coupling direction between neural oscillations. In addition, it should be noted that the present form of the WSTE method cannot directly be applied to more than two neural oscillations, which will be further studied in our future works.

## Figures and Tables

**Table 1:** Physiological interpretation of the model parameters.

Parameter	Interpretation
A	Average excitatory synaptic gain
B	Average inhibitory synaptic gain
$C_{1},C_{2}$	Average number of synaptic contacts in the excitatory feedback loop
$C_{3},C_{4}$	Average number of synaptic contacts in the inhibitory feedback loop
$v_{0},e_{0},r$	Parameters of the nonlinear sigmoid function (transforming the average membrane potential to an average density of action potentials)
$a_{d}$	Average time delay on efferent connection from a population
$K^{i}$	Connectivity constant associated with the connection between populations
